# Coenzyme Q10 protects against hyperlipidemia-induced cardiac damage in apolipoprotein E-deficient mice

**DOI:** 10.1186/s12944-018-0928-9

**Published:** 2018-12-08

**Authors:** Xiaoqing Zhang, Hongyang Liu, Yuhua Hao, Lulu Xu, Tiemei Zhang, Yingshu Liu, Lipeng Guo, Liyue Zhu, Zuowei Pei

**Affiliations:** 1 Department of Infection, Affiliated Zhongshan Hospital of Dalian University, No. 6 Jiefang Street, Dalian, China; 2 Department of Heart Intensive Care Unit, the First Affiliated Hospital of Dalian Medical University, No.193 Lianhe Road, Dalian, China; 3 Department of Endocrinology, Dalian Municipal Central Hospital, 42 Xuegong Road, Dalian, China; 4 Department of Cardiology, Dalian Third People’ Hospital Affiliated to Dalian Medical University, No.40 Qianshan Road, Dalian, China; 5 Rehabilitation Center, Zhejiang Hospital, 12 Lingyin Road, Hangzhou, Zhejiang, China; 6 Department of Cardiology, Affiliated Zhongshan Hospital of Dalian University, No. 6 Jiefang Street, Dalian, China

## Abstract

**Background:**

Hyperlipidemia is a well-established risk factor for cardiac damage, which can lead to cardiovascular diseases*.* Many studies have shown that Coenzyme Q10(CoQ10) protects against cardiac damage in vivo. The aim of this study was to investigate the possible protective effects of CoQ10 against cardiac damage in apolipoprotein E-deficient (ApoE^−/−^) mice.

**Methods:**

Eight-week-old male C57BL/6 and ApoE^−/−^ mice were randomly divided into four groups: C57BL/6 mice fed a normal diet (C57BL/6 group); C57BL/6 mice fed a normal diet + CoQ10 (C57BL/6 + CoQ10 group); ApoE^−/−^ mice fed a high-fat diet (ApoE^−/−^ HD group), and ApoE^−/−^ mice fed a high-fat diet + CoQ10 (ApoE^−/−^ HD + CoQ10 group). All groups were fed the different diets for 16 weeks. Blood samples were obtained from the inferior vena cava and collected in serum tubes. The samples were then stored at − 80 °C until used. Coronal sections of heart tissues were fixed in 10% formalin and then embedded in paraffin for histological evaluation. The remainder of the heart tissues was snap-frozen in liquid nitrogen for mRNA or immunohistochemical analysis.

**Results:**

The metabolic parameters such as total cholesterol (TC), low-density lipoprotein-cholesterol (LDL-c), and triglycerides (TG) levels were lower in ApoE^−/−^HD + CoQ10 mice than in ApoE^−/−^ HD mice. There were significant pathophysiological changes (H&E, PAS, Masson and CD68 staining) in ApoE^−/−^ mice in the HD group compared with those in the HD + CoQ10 group*.* CoQ10 reduced HD-induced cardiac tissue damage via autophagy (p62 and LC3), as evidenced by immunoblotting, immunohistochemistry, and RT-qPCR. CoQ10 also inhibited inflammation (IL-6 and TNF-α) gene expression in ApoE^−/−^ mice.

**Conclusions:**

These results indicate that CoQ10 is a potential therapeutic target for cardiac damage caused by hyperlipidemia.

## Introduction

Cardiovascular disease (CVD) is the primary cause of mortality and morbidity worldwide [1, 2]. The World Health Organisation predicted that nearly 23.6 million people will die from CVD each year by 2030 [3]. Hyperlipidemia is second in a list of the 10 most common chronic conditions, only below hypertension [4]. Hyperlipidemia plays a key role in the onset and progression of CVD. This fact has been verified by many previous studies. Despite hype surrounding the development of new drugs, global CVD and hyperlipidemia are still puzzling. Apolipoprotein E-deficient (ApoE^−/−^) mice have been widely used as models of atherosclerosis because they can develop hyperlipidemia and atherosclerotic lesions similar to those found in humans [5]. Hence, in our study, we established a hyperlipidemia cardiac damage animal model in ApoE^−/−^ mice. Various studies have reported that, in the case of cardiac damage, lipid deposition, inflammatory infiltration, macrophage accumulation, and autophagy have major roles in disease pathogenesis. Zhao et al. observed mononuclear cell infiltration in early lesions, increased expression of inflammatory cytokines and macrophage accumulation in lesions of ApoE^−/−^ mice. In addition, mononuclear cell trafficking and endothelial inflammation affected atherogenesis [6]. Elevated levels of serum interleukin-6 (IL-6) and tumour necrosis factor-α (TNF-α), as inflammation mediators, are closely linked to atherosclerosis [7].

Coenzyme Q10 (CoQ10), referred to as ‘ubiquinone’, was discovered in 1957. It is comprised of a lipophilic benzoquinone structure with a side chain of 10 isoprenoid units [8, 9]. CoQ10 is a critical intermediate of mitochondrial calcium-dependent ion channels for the synthesis of adenosine triphosphate (ATP) [10]. Previous research has identified that the biological importance of CoQ10 is related to its antioxidant activity, free radical scavenging, and restoration of the antioxidant defence system [11, 12]. A number of studies have shown that CoQ10 can ameliorate acute myocardial ischemia-reperfusion injury [13], improve heart function [14], and decrease cardiovascular mortality [15]. Anayt et al. found that CoQ10 prevents isoprenaline-induced cardiac remodelling in aged rats. Histopathological examination of heart tissue revealed focal areas of endocardium degeneration, mononuclear cell infiltration, fibrous tissue deposition, and increased thickness of the myocardium of the left ventricle [16]. There is no published research that has investigated the role of exogenous CoQ10 in hyperlipidemia-induced cardiac damage in ApoE^−/−^ mice.

Therefore, in our study, we aimed to determine whether, and by what mechanism, CoQ10 can protect against hyperlipidemia-induced cardiac damage in ApoE^−/−^ mice.

## Experimental

### Animals and drug treatment

Six-week old male C57BL/6 and ApoE^−/−^ mice were obtained from Beijing Vital River Laboratories Animal Technology Co., Ltd. (Beijing, China). All mice were housed in a room with a 12/12-h light-dark cycle at a controlled temperature (24 °C). Male C57BL/6 mice were randomly divided into two groups as follows: mice fed a normal diet (C57BL/6 group, *n* = 10), and mice fed a normal diet + CoQ10 (100 mg/kg/day, Sigma-Aldrich, St. Louis, MO, USA) (C57BL/6 + CoQ10 group, *n* = 10). Male ApoE^−/−^ mice were randomly divided into two groups as follows: mice fed a high-fat diet (ApoE^−/−^ HD group, n = 10), and mice fed a high-fat diet + CoQ10 (100 mg/kg/day) (ApoE^−/−^ HD + CoQ10 group, n = 10). The high-fat diet consisted of a commercially prepared mouse food (MD12017) supplemented with 20.0% (wt/wt) coco fat, 1.25% (wt/wt) cholesterol, 22.5% (wt/wt) protein, and 45.0% carbohydrate (Jiangsu Mediscience Ltd., Jiangsu, China). All groups were fed different diets for 16 weeks. Blood samples were obtained from the inferior vena cava, collected in serum tubes, and stored at − 80 °C until use. Longitudinal sections of the hearts were fixed in 10% formalin and embedded in paraffin for histological evaluation. The remainder of the heart tissue was snap-frozen in liquid nitrogen for mRNA isolation and immunoblotting analyses. All animal experiments were performed in accordance with the Guide for the Care and Use of Laboratory Animals. The study was approved by the ethics committee of the affiliated Zhongshan Hospital of Dalian University of China..

### Blood biochemistry

Serum concentrations of total cholesterol (TC), low-density lipoprotein cholesterol (LDL-c), and triglyceride (TG) were measured using an ELISA kit (Westang, Shanghai, China).

### Haematoxylin and eosin staining

Cardiac tissues were fixed in 10% buffered formalin solution for 30 min, dehydrated in 75% ethanol overnight, and then embedded in paraffin. Serial sections (4-μm-thick) were subjected to haematoxylin and eosin (H&E) staining for assessment of pathological changes by microscopy.

### Periodic acid-Schiff staining

Cardiac tissues from each group were stored in 10% formalin, dehydrated in an ascending alcohol series (75, 85, 90, and 100% alcohol, 5 min each), and then embedded in paraffin wax. Paraffin sections (4-μm-thick) were sliced from these paraffin-embedded tissue blocks. Tissue sections were then de-paraffinised via immersion in xylene (3 times, 5 min each) and rehydrated using a descending alcohol series (100, 90, 85, and 75% alcohol, 5 min each). Biopsy samples were stained using Periodic acid-Schiff (PAS) stain to investigate changes in cardiac morphology and fibrosis. Red staining indicated lipid deposition.

### Masson’s trichrome staining

Cardiac tissue from each group was stored in 10% formalin, dehydrated in an ascending alcohol series (75, 85, 90, and 100% alcohol, 5 min each), and embedded in paraffin wax. Paraffin sections (4-μm-thick) were sliced from these paraffin-embedded tissue blocks. Tissue sections were de-paraffinised via immersion in xylene (3 times, 5 min each) and rehydrated using a descending alcohol series (100, 90, 85, and 75% alcohol, 5 min each). Biopsy samples were stained using Masson’s trichrome stain to investigate heart morphological and fibrotic changes. Blue staining indicated collagen accumulation. The results were visualised using an Olympus microscope (Olympus, Tokyo, Japan).

### RNA isolation and real-time-PCR

Total RNA was isolated from cardiac tissues, using ISOGEN reagent (Nippon Gene, Tokyo, Japan) according to the manufacturer’s protocol. Complementary DNA (cDNA) was synthesised from total RNA, using a first-strand cDNA synthesis kit (SuperScript VILO cDNA Synthesis Kit; Life Technologies, Carlsbad, CA, USA) according to the manufacturer’s protocol. Gene expression was analysed quantitatively by RT-qPCR, using fluorescent SYBR Green technology (Light Cycler; Roche Molecular Biochemicals). To normalise the relative amounts of target genes, β-actin cDNA was amplified and quantified in each cDNA preparation. Primer sequences are listed in Table 1.

**Table 1:** Primer oligonucleotide sequences

Gene	Primers
P62	F: 5′-TCCCAATGTCAATTTCCTGAAGA-3′
	R: 5′- TCTGTGCCTGTGCTGGAACT-3′
LC3	F: 5′-AGCTGCCTGTCCTGGATAAGAC-3′
	R: 5′- GGTGTGGAGACGCTCACCAT-3′
IL-6	F:5′-TACCAGTTGCCTTCTTGGGACTGA-3′
	R:5′-TAAGCCTCCGACTTGTGAAGTGGT-3′
TNF-α	F:5′-TCTCATGCACCACCATCAAGGACT-3′
	R:5′-ACCACTCTCCCTTTGCAGAACTCA-3′
β-actin	F:5′-CGATGCCCTGAGGGTCTTT-3′
	R:5′-TGGATGCCACAGGATTCCAT-3′

Abbreviations: *IL-6* interleukin- 6, *TNF-α* tumor necrosis factor-α

### Western blot analysis

Cardiac tissues were harvested, and protein extracts were prepared according to established methods. The extracts were separated by sodium dodecyl sulphate-polyacrylamide gel electrophoresis (SDS-PAGE, 8–15%) and transferred to a polyvinylidene difluoride (PVDF) membrane (Millipore, Bedford, MA, USA). The membranes were blocked with 5% milk and incubated with the indicated primary antibodies at 4 °C overnight. Primary antibodies against LC3 (rabbit anti-LC3 antibody, 1:1000; Proteintech, Wuhan, China), p62 (rabbit anti-p62 antibody, 1:1000; Proteintech), anti-β-actin (1:1000; Cell Signaling Technology), phospho-ERK (Rabbit anti-phospho-ERK, 1:1000; Cell Signaling Technology), and total ERK (Rabbit anti-total ERK,1:1000; Cell Signaling Technology) were used. After washing, the membranes were incubated with the appropriate secondary antibodies. The membranes were exposed to enhanced chemiluminescence-plus reagents (Beyotime Institute of Biotechnology, Hangzhou, China). This experiment was carried out in triplicate. Emitted light was captured by a Bio-Rad imaging system with Chemi HR camera 410 and analysed with Gel-Pro Analyzer version 4.0 (Media Cybernetics, Rockville, MD, USA). This analysis was carried out independently three times. Protein levels are expressed as protein/β-actin ratios to minimise loading differences. The relative signal intensity was quantified using NIH ImageJ software.

### Immunohistochemistry

Hearts were dissected free from the surrounding connective tissue, and fixed with 4% paraformaldehyde, embedded in paraffin, and then cut into slices using a microtome (Leica RM 2235 or Leica CM1850UV; Leica, Solms, Germany). The slices were then mounted onto glass slides, and histological examinations were performed. Immunohistochemistry was performed using Histofine Simple Stain kit (Nichirei, Tokyo, Japan), according to the manufacturer’s instructions. Briefly, sections were deparaffinised with xylene and then rehydrated in a descending ethanol series. Sections were treated with 3% H_2_O_2_ in methanol for 15 min to inactivate endogenous peroxidases and then incubated with a primary antibody against p62 (rabbit anti-p62 antibody, 1:200; Proteintech); LC3 (rabbit anti-LC3 antibody, 1:200; Proteintech); CD68 (rabbit anti-CD68 antibody, 1:250; Abcam) at room temperature for 1 h. All sections were examined under an Olympus BX40 upright light microscope (Olympus, Tokyo, Japan).

### Statistical analysis

All data are presented as the mean ± SEM. Statistical analysis was performed using SPSS software version 23.0. Inter-group variation was measured by one-way ANOVA followed by Tukey’s test. The minimal level of significance was *P* < 0.05.

## Results

### Metabolic characterisation

According to the metabolic characteristics, we found the results of serum lipid measurements **(**Table 2**)** indicated that a hyperlipidemia mouse model had been successfully established. Body weights (BWs) did not differ among the four groups. The ApoE^−/−^HD mice group showed markedly increased TC, TG and LDL-c levels, but these were significantly decreased in the HD + CoQ10 group. These results indicate that CoQ10 decreased TC, TG and LDL-c in the ApoE^−/−^HD mice.

**Table 2:** Metabolic data from the four groups after 16 weeks of different treatment

	C57BL/6 (*n* = 10)	C57BL/6 + CoQ10 (*n* = 10)	ApoE^−/−^ HD (*n* = 10)	ApoE^−/−^ HD + CoQ10 (*n* = 10)
BW (mg)	24.53 ± 2.03	25.32 ± 1.96	31.31 ± 3.51	28.55 ± 1.16
TC (mmol/L)	8.72 ± 0.20**	7.58 ± 0.17**	27.20 ± 2.68	12.21 ± 2.87*
TG (mmol/L)	0.48 ± 0.08**	0.53 ± 0.04**	2.29 ± 0.18	1.17 ± 0.09*
LDL-c (mmol/L)	7.60 ± 2.24**	7.10 ± 2.83**	24.62 ± 0.77	9.70 ± 0.2**

Abbreviations: *BW* body weight, *TC* total cholesterol, *TG* triglycerides, *LDL-c* low-density lipoprotein cholesterol

Data are means ± SEM; *n* = 10 per group. * *P* < 0.05 vs ApoE^−/−^HD; ** *P* < 0.01 vs ApoE^−/−^HD

### Histopathological changes in cardiac tissues

**Fig. 1:**
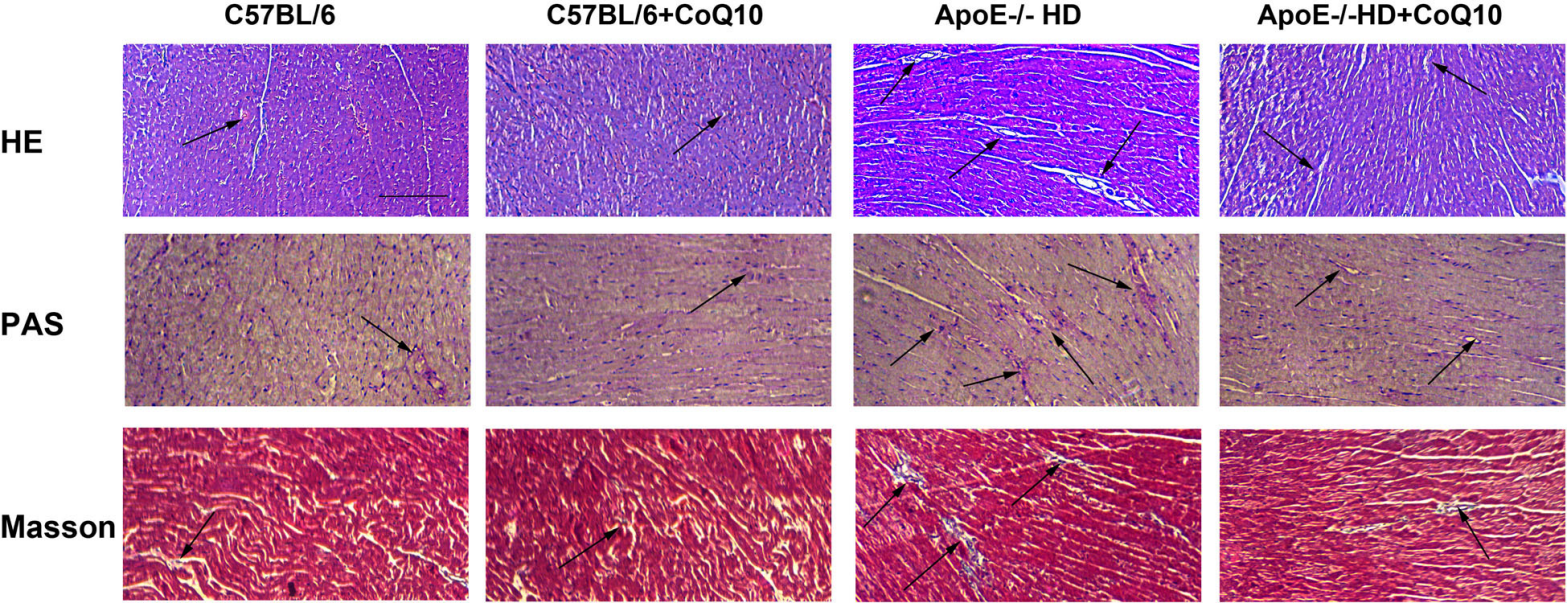
Effect of CoQ10 on hyperlipidemia-induced histopathological changes in cardiac tissues. Histopathological changes were evaluated by H&E, PAS, and Masson’s trichrome staining in the cardiac tissue of mice with different treatment (*n* = 5). Scale bar = 100 μm. The arrows indicate damage

To evaluate cardiac tissue damage, we used the HE, PAS, and Masson’s trichrome staining facilitated the visualisation of cardiac structural disorder, inflammatory cell infiltration, massive fibrosis, and collagen deposition with cardiac damage as seen in hyperlipidemia. Treatment with CoQ10 significantly ameliorated inflammatory cell infiltration, fibrosis, and collagen deposition in ApoE^−/−^ HD + Q10 group compared to that in ApoE^−/−^ HD group (Fig. 1). These results indicate that CoQ10 can reduced cardiac tissue damage in the ApoE^−/−^HD mice.

### CoQ10 **reduced tumour necrosis factor (TNF)-α and interleukin (IL)-6 gene expression in cardiac tissue of ApoE**^**−/−**^**mice fed HD**

**Fig. 2:**
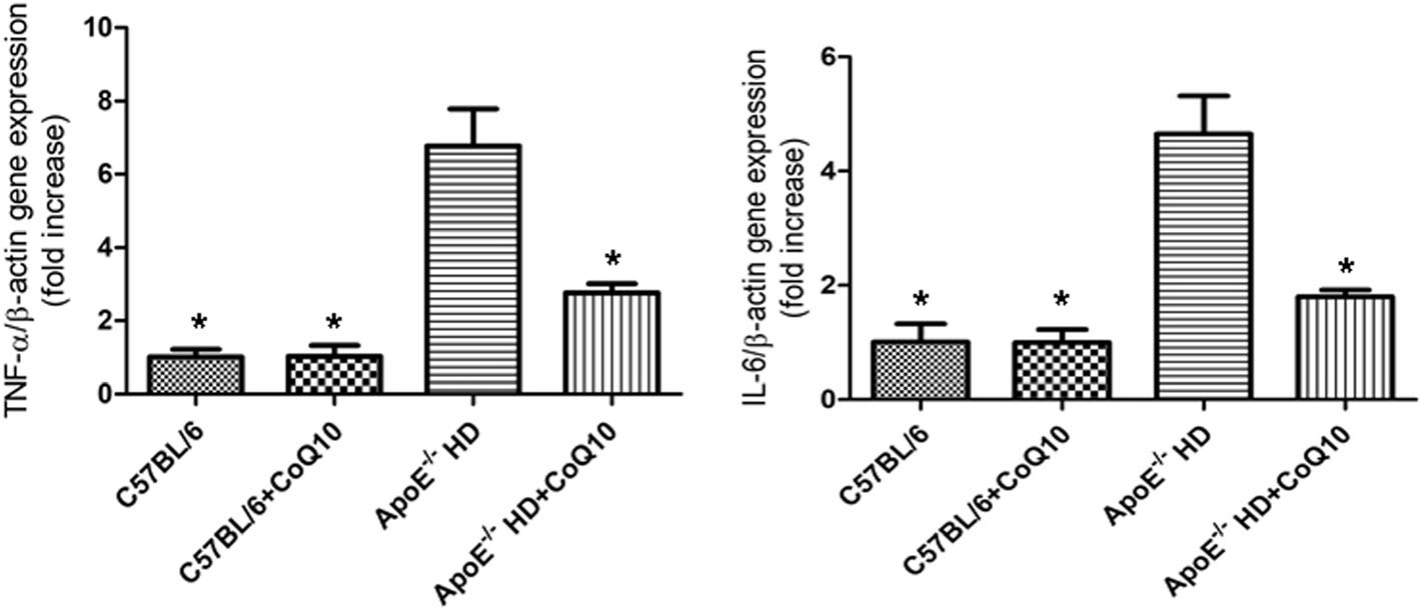
Pro-inflammatory gene expression in the cardiac tissue of the four groups of mice after 16 weeks on different diets. Relative mRNA expression of *TNF-α* and *IL-6* in the cardiac tissue of each group after 16 weeks under different treatments. Data are given as the means ± SEM; *n* = 6 in each group. * *P* < 0.01 vs. ApoE^−/−^ HD

To examine the involvement of pro-inflammatory cytokines in hyperlipidemia-induced cardiac damage, *IL-6* and *TNF-α* gene expression was measured by RT-q PCR (Fig. 2). Both *IL-6* and *TNF-α* were up-regulated in ApoE^−/-^HD mice. However, this up-regulation was attenuated in ApoE^−/-^HD + CoQ10 mice (*p* < 0.01). These results indicate that CoQ10 inhibited pro-inflammatory cytokines expression in the ApoE^−/−^HD mice.

### CoQ10 **reduced macrophage numbers in cardiac tissue of ApoE**^**−/−**^**mice fed HD**

**Fig. 3:**
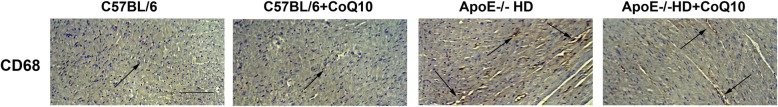
CD68 expression in cardiac tissues of the four groups after 16 weeks under different treatments. Representative immunohistochemical staining for CD68 expression in cardiac tissue of mice with different treatment (*n* = 5). Scale bar = 100 μm. Arrows indicate positively stained cells

To detect infiltrating macrophages expression in the different treament group mice, immunohistochemical analysis using CD68 was performed (Fig. 3). The mice in the HD + CoQ10 group exhibited markedly reduced CD68-positive staining in the cardiac tissue compared to the ApoE^−/−^HD mice. These results indicated that CoQ10 reduced macrophage infiltration in the ApoE^−/−^ HD mouse hearts.

### CoQ10 **reduced p62 and increased LC3 expression in cardiac tissues**

**Fig. 4:**
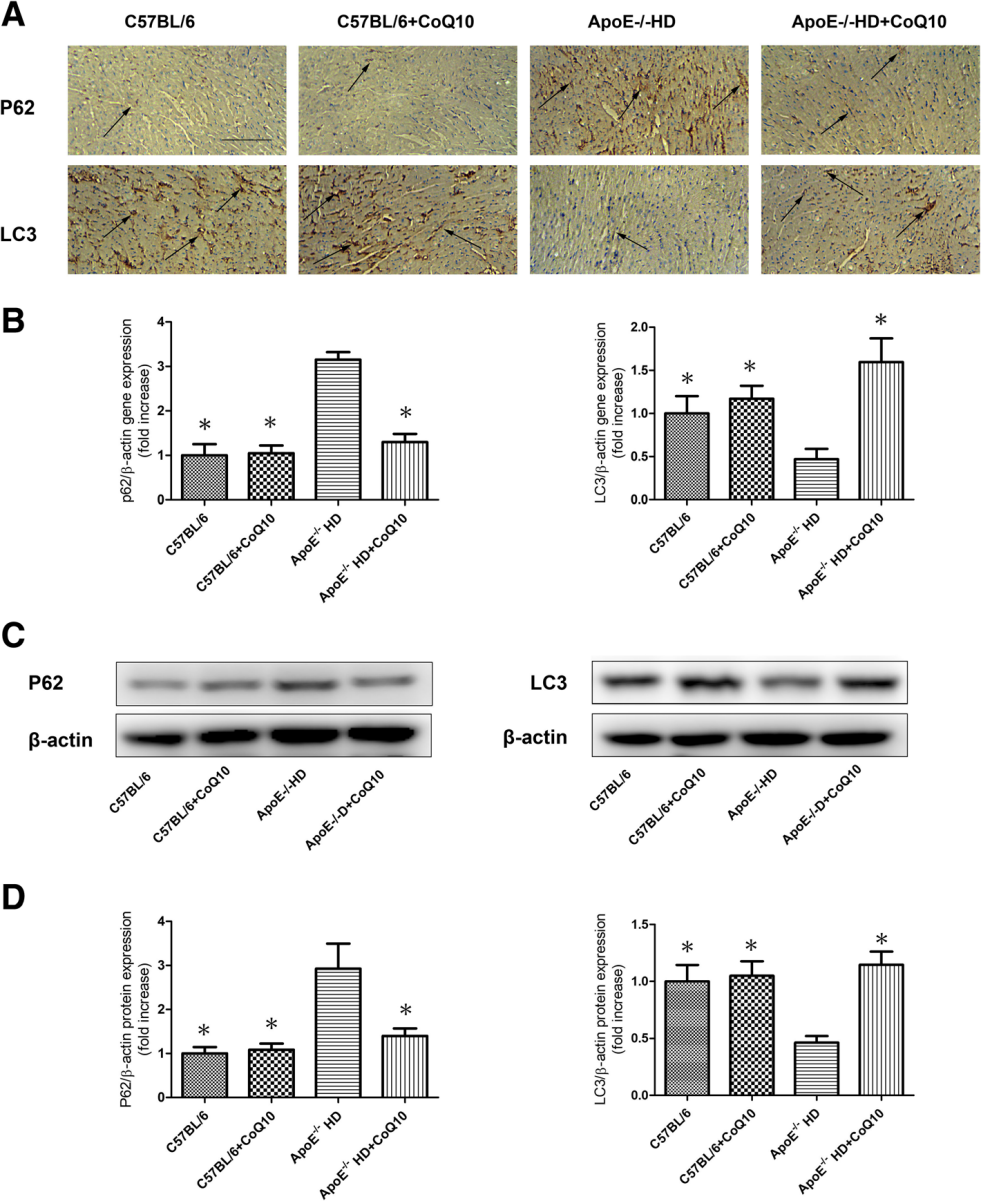
P62 and LC3 expression in cardiac tissues of the four groups after 16 weeks under different treatments. **a** Representative immunohistochemistry for p62 and LC3 in cardiac tissues. Scale bar = 100 μm. Arrows indicate positively stained cells. **b** Relative mRNA expression of *p62* and *LC3* in cardiac tissue of each group after 16 weeks under different treatments. **c** Immunoblotting for p62 0061nd LC3 in cardiac tissues. **d** Bar graph showing quantification of p62 and LC3 protein expression. Data are given as the means ± SEM; *n* = 5–6 in each group. * *P* < 0.05 vs. ApoE^−/−^ HD

To evaluate p62 and LC3 expression in the cardiac tissues, p62 and LC3 immunostaining were performed (Fig. 4a). The HD + CoQ10 group had markedly reduced p62 and increased LC3 expression in cardiac tissues compared to the HD groups. RT-qPCR was performed for *p62* and *LC3* gene expression (Fig. 4b). We found that *p62* gene expression was significantly suppressed and *LC3* gene expression significantly increased in the HD + CoQ10 group, compared with that in the HD group. Immunoblotting was performed for p62 and LC3 proteins (Fig. 4c). We found that p62 expression was significantly suppressed and LC3 expression significantly increased in the HD + CoQ10 group, compared with that in the HD group. (Fig. 4d). These results indicated that CoQ10 reduced p62 and increased LC3 expression in ApoE^−/−^ HD mice.

### CoQ10 **reduced phospho-ERK levels in cardiac tissues of ApoE**^**−/−**^**mice fed HD**

**Fig. 5:**
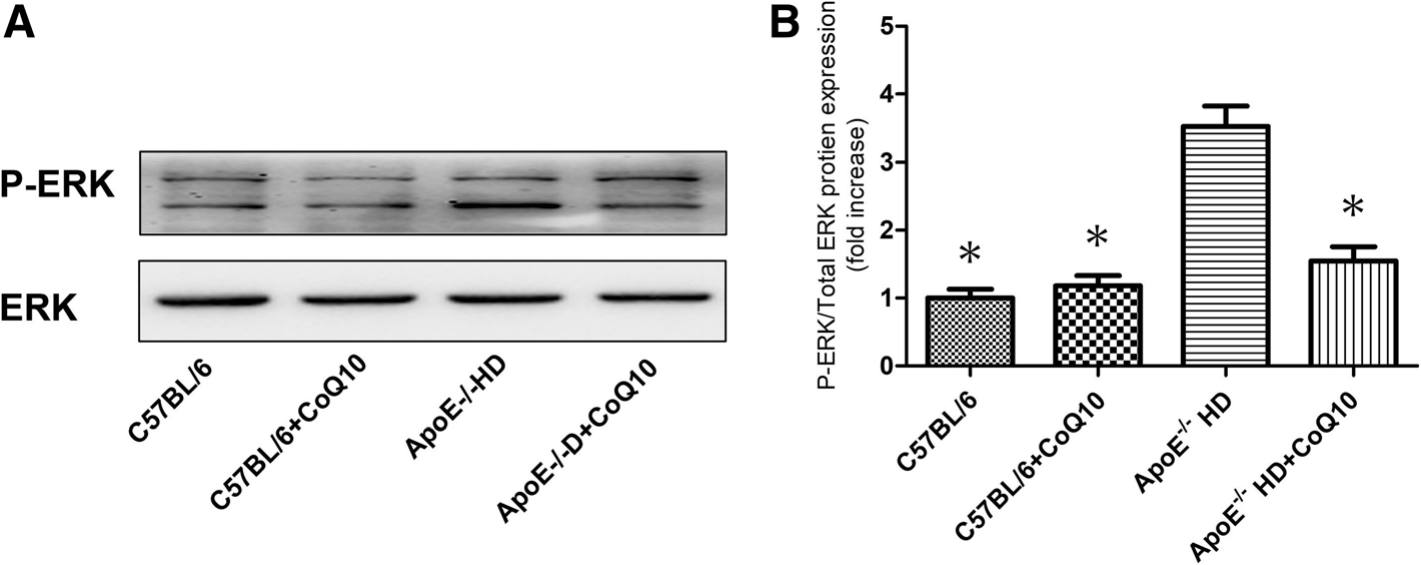
Phospho-ERK expression in the cardiac tissues of the four groups after 16 weeks under different treatments. **a** Immunoblotting for phospho-ERK and total ERK levels expression in cardiac tissues. **b** Bar graph shows the quantification of phospho-ERK/total ERK protein levels. Data are given as the means ± SEM; *n* = 3 in each group. *P < 0.05 vs ApoE^−/−^HD

Protein kinases play a role in autophagy, to analyse the phosphorylation of ERK, p-ERK and total ERK protein immunoblotting were performed (Fig. 5a). We found that the phosphorylation level of ERK in HD + CoQ10 mice was significantly suppressed compared to that in ApoE^−/−^HD mice (Fig. 5b). These results indicated that CoQ10 decreased phospho-ERK protein expression in ApoE^−/−^ HD mice.

## Discussion

This study demonstrated that CoQ10 has a protective effect against cardiac damage via pro-inflammatory cytokine, macrophage accumulation and autophagy in hyperlipidemia.

With respect to the metabolic characteristics, we found that TC, TG, and LDL-c levels were elevated in the HD-fed mice, compared with the other groups mice. These results are in agreement with those reported by Kolbus and colleagues [17]*.* Interestingly, TC, TG, and LDL-c were significantly suppressed in the HD + CoQ10 group compared to that in the HD group. Previous studies have demonstrated that supplementation with CoQ10 significantly inhibits low-density lipoprotein oxidation [18]. Our results indicated that CoQ10 influences cholesterol metabolism. CoQ10 inhibits the peroxidation of LDL-c which may play a key role in its anti-atherogenic effects and reduced cardiac damage [19]. These results indicate that CoQ10 influences the cholesterol metabolisms. However, further studies are needed to clarify the mechanisms involved. The H&E, PAS, and Masson staining results revealed increased leukocyte infiltration, lipid deposition, and collagen accumulation in the HD-fed mice compared to those in the CoQ10 + HD group. These results indicate that CoQ10 can reduced cardiac tissue damage in the ApoE^−/−^HD mice. Taken together, serum and histological results confirmed that cardiac damage occurred in the HD-fed mice, but this damage was significantly suppressed in the CoQ10 + HD group.

CoQ10 has been found to be a key component in mitochondrial function [20]. Localized in the inner mitochondrial membrane, it facilitates electron transfer in the generation of adenosine triphosphate (ATP) [21]. It has also been shown that CoQ10 has anti-oxidant and anti-inflammatory effect, preventing the oxidation of proteins, lipids deposition [22].

Pro-inflammatory genes (*TNF-α* and *IL-6*) have been reported to be expressed at high levels and contribute to cardiac damage in hyperlipidemia [23, 24]*.* The present study showed that *TNF-α* and *IL-6* gene expression was reduced in the HD + CoQ10 group compared to that in the HD-fed group. This indicates that CoQ10 supressed *TNF-α* and *IL-6* gene expression in HD-fed mice.

Cardiac damage induced by hyperlipidemia is usually associated with an increase in the number of macrophages. Macrophage-derived foam cells release cytokines that recruit more macrophages to lesions and influence lipid deposition [25]. The marker CD68 identifies macrophages, and CD68-positive cells were found in liver tissue damaged by hyperlipidemia [26]*.* In the present study, immunohistochemical staining with anti-CD68 antibody showed that the number of CD68-positive cells significantly increased in the HD-fed group compared to that in the C57BL/6 and C57BL/6 + CoQ10 group. However, the HD + CoQ10 group showed markedly reduced accumulation of CD68-positive cells in heart tissue compared to the HD-fed group. This indicates that CoQ10 reduced macrophage accumulation in HD-fed mice, furthermore, CoQ10 inhibied foam cell formation and lipid accumulation.

Autophagy is a self-renewal pathway that mediates the degradation of cytoplasmic contents in lysosomes, thus maintaining cellular metabolic homeostasis [27, 28]. Insufficient autophagy can promote programmed cell death, apoptosis [29], which results in cardiac damage [30]. It has been reported that LC3 and p62 are central autophagy-related proteins involved in the autophagy flux [31–33]. LC3, a mammalian ortholog of yeast Atg8 (autophagy-related gene products), is a ubiquitin-like protein that becomes lipidated and tightly associated with autophagosomal membranes [34, 35]. p62, as a LC3-interacting protein, transports ubiquitinated protein aggregates to autophagosomes [36]. When autophagy is impaired, p62 levels increase in cells and tissues [37]. Previous studies showed that hyperlipidemia increases cardiac p62 level and decreases LC3 expression in vivo [38, 39]. In an earlier study, CoQ10 supressed p62 and increased LC3 expression in cardiac tissue with hyperlipidemia.

Protein kinases regulate autophagy and, as shown earlier, phosphorylation of ERK regulates p62 and LC3 expression both in vivo and in vitro [40–42]. Our results showed that phosphorylation of ERK was significantly reduced in the HD + CoQ10 group compared to that in the HD-fed group. We speculate that CoQ10 regulates p62 and LC3 expression via the phospho-ERK pathway, furthermore, CoQ10 reduced cardiac damage according to phospho-ERK pathway.

## Conclusions

Our study established that CoQ10 contributes to the mitigation of hyperlipidaemic cardiac damage, as shown by the downregulation of lipid deposition, pro-inflammatory gene expression, macrophage accumulation, and autophagy upregulation. These findings provide new insights into the role of CoQ10 in hyperlipidemia-induced cardiac damage and raise the possibility of a novel therapeutic intervention for treatment of CVDs.

## Abbreviations

ApoE^−/−^ mice: apolipoprotein E-deficient mice
CoQ10: Coenzyme Q10
IL-6: interleukin- 6
LDL-c: low-density lipoprotein cholesterol
TC: total cholesterol
TG: triglycerides
TNF-α: tumor necrosis factor-α
